# Cleaner Production of Metallurgical-Grade Iron from High-Iron Bauxite Residue via Smelting Reduction: Thermodynamic Control, Industrial Application Potential, and Slag Utilization Strategy

**DOI:** 10.3390/ma18143288

**Published:** 2025-07-11

**Authors:** Kun Wang, Ting-An Zhang, Zhi-He Dou, Yan Liu, Guo-Zhi Lv

**Affiliations:** 1 School of Metallurgy, Northeastern University, Shenyang 110819, China; wangk@smm.neu.edu.cn (K.W.); douzh@smm.neu.edu.cn (Z.-H.D.); liuyan@smm.neu.edu.cn (Y.L.); lvgz@smm.neu.edu.cn (G.-Z.L.); 2 Key Laboratory of Ecological Metallurgy of Multi-Metal Intergrown Ores of Ministry of Education, Shenyang 110819, China

**Keywords:** high-iron bauxite residue, pig iron, smelting reduction, extraction and recovery, secondary resource

## Abstract

Iron-rich bauxite residue (red mud) is a hazardous alkaline solid waste produced during the production of alumina from high-iron bauxite, which poses severe environmental challenges due to its massive stockpiling and limited utilization. In this study, metallic iron was recovered from high-iron red mud using the smelting reduction process. Thermodynamic analysis results show that an increase in temperature and sodium oxide content, along with an appropriate mass ratio of Al_2_O_3_ to SiO_2_ (A/S) and mass ratio of CaO to SiO_2_ (C/S), contribute to the enhancement of the liquid phase mass fraction of the slag. During the smelting reduction process of high-iron red mud, iron recoveries for low-alkali high-iron red mud and high-alkali high-iron red mud under optimal conditions were 98.14% and 98.36%, respectively. The metal obtained through reduction meets the industrial standard for steel-making pig iron, which is also confirmed in the pilot-scale experiment. The smelting reduction process of high-iron red mud can be divided into two stages, where the reaction is predominantly governed by interfacial chemical reaction and diffusion control, respectively. The apparent activation energy of high-alkali high-iron red mud is lower than that observed for low-alkali high-iron red mud. The reduced slag can be used as a roadside stone material or cement clinker. This proposed method represents a sustainable process for the comprehensive utilization of high-iron red mud, which also promotes the minimization of red mud.

## 1. Introduction

Currently, over 90% of global alumina is produced using the Bayer process [1]. During the digestion process, a strongly alkaline bauxite residue (red mud) is produced, with the primary phase being hydrated sodium aluminosilicate (Na_2_O·Al_2_O_3_·*x*SiO_2_·nH_2_O) formed by Na_2_O, Al_2_O_3_, and SiO_2_ [2,3,4]. Typically, 1–1.5 tons of red mud is discharged per ton of alumina [5,6]. In 2023, China’s alumina output reached 82.51 million tons according to the China Statistical Yearbook 2024 compiled by the National Bureau of Statistics of China (NBSC), generating massive red mud [7]. China’s accumulated red mud stockpile now exceeds 1.3 billion tons, with annual discharge over 100 million tons [8]. The leachate of red mud is strongly alkaline, with the pH values greater than 12 [9]. Due to this alkalinity, most red mud is stockpiled or landfilled, occupying land and causing soil alkalization, water pollution, and risks like collapses and landslides [10,11]. Therefore, the environmental issues caused by the large-scale accumulation of red mud have become increasingly serious in recent years [12].

The current comprehensive utilization of red mud mainly focuses on construction materials, agriculture, environmental remediation, and valuable metal extraction [13]. However, its strong alkalinity limits large-scale industrial promotion and application [14]. Iron in red mud mainly exists as hematite (Fe_2_O_3_), with minor quantities of goethite (FeOOH) [15]. Depending on the raw material sources, the Fe_2_O_3_ content in red mud varies from 6% to 60% [16]. High-iron red mud (35–50% iron-bearing phase) is recognized as a potential multi-metal source dominated by iron, serving as a critical secondary resource for strategic metal recovery [17,18]. Global research on high-iron red mud mainly focuses on iron recovery via three key techniques: physical magnetic separation, solid-phase reduction combined with magnetic separation, and smelting reduction processes [19,20]. The physical magnetic separation method utilizes high-intensity magnetic separators to process high-iron red mud using the weak magnetism of hematite to effectively separate the iron component [21]. However, direct magnetic separation efficiency is limited due to iron oxides being wrapped by gangue minerals [22]. The solid-phase reduction combined with magnetic separation for iron recovery involves mixing high-iron red mud with carbonaceous reductants and subsequent reduction roasting at elevated temperatures [23]. This thermal treatment promotes the carbothermic reduction of hematite (Fe_2_O_3_) to magnetite (Fe_3_O_4_), enhancing magnetism and achieving a 90% iron recovery rate [24,25]. However, the formation of FeO·2Al_2_O_3_ and 2FeO·2Al_2_O_3_ compounds during solid-phase reduction leads to high alumina content in the iron concentrate [26].

Recent pyrometallurgical studies show that iron oxide in high-iron red mud can be effectively reduced to metallic iron via smelting reduction, with high iron recovery efficiency [27]. However, the traditional blast furnace is unable to handle high-iron red mud. That is because red mud contains significantly higher Al_2_O_3_, SiO_2_, and other oxides than conventional iron ores, leading to large slag volume and high energy consumption in blast furnace smelting [28].

**Figure 1:**
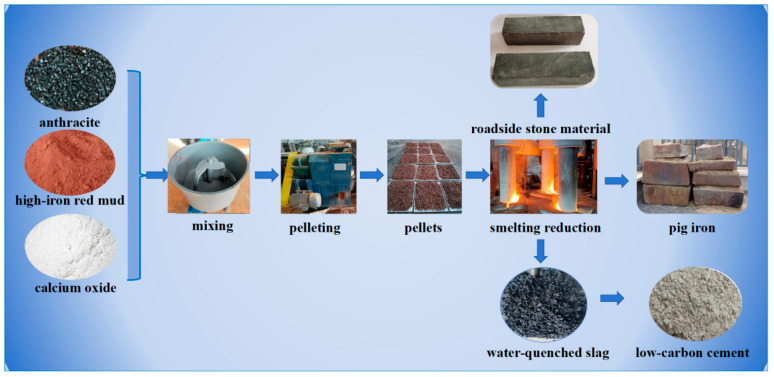
Process of high-efficiency preparation of iron from high-iron red mud.

To extract the metallurgical-grade iron from high-iron red mud and fully utilize reduction slag, a novel smelting reduction method was proposed [29], the process flow chart is shown in Figure 1. This study systematically explored the liquid phase mass fraction, liquidus, reduction behavior, and kinetic mechanisms of the Al_2_O_3_-SiO_2_-CaO-Na_2_O system during smelting reduction using a combined thermodynamic theoretical and high-temperature experimental approach. Finally, a pilot-scale experiment was conducted to establish a theoretical foundation for the industrial-scale, zero-waste, and high-value comprehensive utilization of high-iron red mud, bridging the gap between lab research and industrial application.

## 2. Materials and Methods

### 2.1. Raw Materials

The high-iron red mud used in this study was obtained from Shandong Province’s alumina plants, with chemical compositions presented in Table 1. Specifically, the low-alkali high-iron red mud (from Weiqiao Shandong) features low alkali and high iron, while the high-alkali high-iron red mud (from Xinfa Shandong) has high alkali and iron contents. Table 1 shows that the low-alkali sample contains 53.71% iron oxide and 3.02% sodium oxide, whereas the high-alkali sample has slightly lower iron oxide (41.63%) but higher alkali (6.48%).

In this study, anthracite (with 83.80% fixed carbon and 5.53% ash) serves as the reducing agent. The ash mainly contains alumina, silicon oxide, calcium oxide, and iron oxide. Given its low content, the impact of ash on the reduction process is disregarded in this experiment. Additionally, pure calcium oxide and calcium fluoride reagents are added as additives to adjust the viscosity and fluidity of the slag.

**Figure 2:**
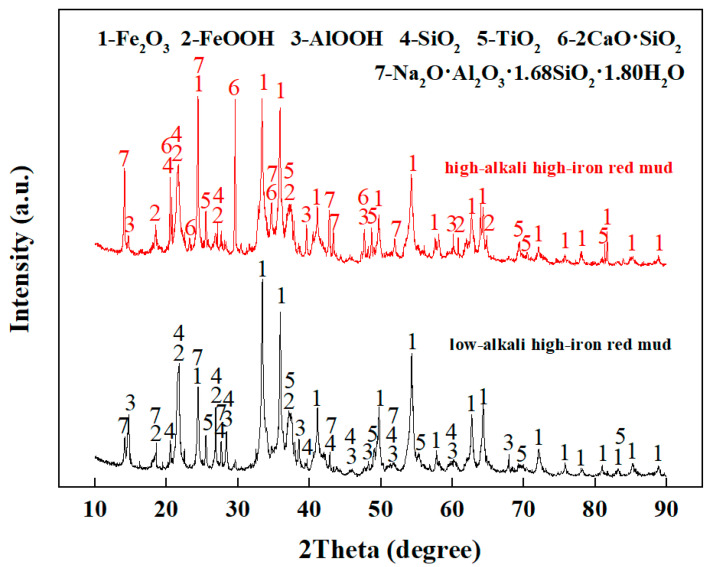
XRD pattern of high-iron red mud.

The XRD (D8 Advance, German Bruker Company, Freiburg, Germany) analysis of high-iron red mud is shown in Figure 2. The main phases identified in both low-alkali and high-alkali high-iron red mud include hematite (Fe_2_O_3_), goethite (FeO(OH)), boehmite (AlO(OH)), quartz (SiO_2_), rutile (TiO_2_), and hydrated sodium aluminosilicate (Na_2_O·Al_2_O_3_·1.68SiO_2_·1.80H_2_O).

**Figure 3:**
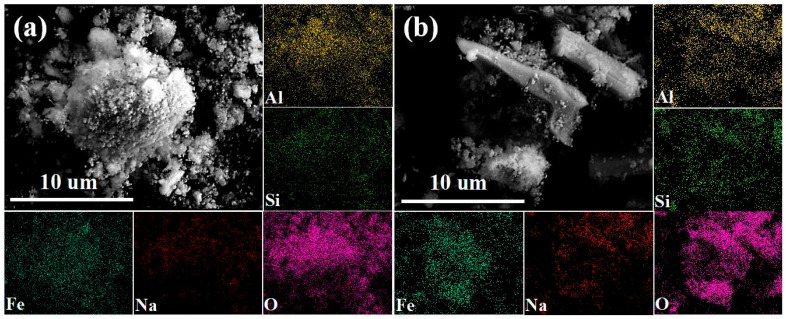
Scanning electron microscopic image of low-alkali high-iron red mud (**a**) and high-alkali high-iron red mud (**b**).

Figure 3 presents the SEM-EDS (SU-8010, Hitachi Corporation of Japan, Tokyo, Japan) analysis results of low-alkali and high-alkali high-iron red mud samples. Both samples show agglomerated particles with porous, loose structures and small particle sizes (approximately 10 μm in diameter). The results of the elemental surface scanning reveal that aluminum, silicon, iron, oxygen, and sodium are the primary elements in these red mud samples.

### 2.2. Experimental Methods

Firstly, high-iron red mud was dried at 150 °C for 12 h. Then, 80 g of dried low-alkali and high-alkali high-iron red mud were respectively weighed, mixed with anthracite, calcium oxide, and calcium fluoride in different proportions. The mixture was placed into a 40mm-diameter stainless steel mold and pressed at 20 MPa for 30 s using a pressing machine to form cylindrical specimens. The smelting reduction experiment was carried out in a vertical tubular resistance furnace, the model of which was RTW-10 (Northeastern University, Shenyang, China). An alumina crucible (Φ 50 mm × 120 mm) was placed in the vertical tubular resistance furnace. After the vertical resistance furnace was powered on, a heating program with a heating rate of 10 °C·min^−1^ was initiated. After reaching the predetermined temperature, the cylindrical samples were carefully placed into the alumina crucible with argon introduced as a protective gas. Reactions proceeded at constant temperature for the set duration, after which the alumina crucible was removed and cooled to room temperature. After the alumina crucible and reduction product had fully cooled, the metal was separated from the reduction slag, followed by precise measurement of the metal and slag. Samples of the metal lump and reduction slag were then collected for detailed chemical and phase composition analysis.

In kinetic experiments, 30 g each of low-alkali and high-alkali high-iron red mud samples were weighed, then CaO was added to adjust the basicity. After mixing, the mixture was compressed into Φ40 mm × 50 mm blocks under 20 MPa using a stainless steel mold. High-purity graphite crucibles were placed in the furnace tube. The vertical resistance furnace was started and programmed to heat from 20 °C to 1200 °C at 10 °C·min^−1^, then reduce the rate to 8 °C·min^−1^ above 1200 °C until reaching the predetermined temperature. The entire process was under argon protection to prevent carbon loss. Once preheated, the sample block was quickly placed into the crucible with tongs to start timing. After reaction, the crucible was removed, and the melt was cast into a graphite mold for air cooling. After cooling, slag-metal separation was performed, and both reduced metal and reduction slag were weighed. Chemical composition analysis calculated iron recovery, while XRD characterized the reduction slag under different conditions.

### 2.3. Analysis Methods

Sample chemical compositions were analyzed via X-ray fluorescence spectrometer (ZSX Primus IV, Rigaku Corporation, Tokyo, Japan), and phases were determined by X-ray diffractometry (D8 Advance, German Bruker Company, Freiburg, Germany). This study investigated the effects of experimental parameters on iron recovery during the smelting reduction of high-iron red mud. The iron recovery rate was calculated using Equation (1).

(1) $$\eta_{\mathrm{Fe}}=\frac{M\alpha_{\mathrm{Fe}}-M_{\mathrm{slag}}\beta_{\mathrm{Fe}}}{M\alpha_{\mathrm{Fe}}}\times 100\%$$

where *η*_Fe_ is the iron recovery rate, %; *M* is the mass of the raw material, g; *M*_slag_ is the mass of the reduction slag, g; *α*_Fe_ is the mass fraction of iron in the raw material, wt.%; *β*_Fe_ is the mass fraction of iron in the reduction slag, wt.%.

## 3. Thermodynamic Analysis

### 3.1. Calculation of Gibbs Free Energy Changes

High-iron red mud primarily contains iron oxide, alumina, silicon oxide, titanium oxide, and sodium oxide. Potential reactions in the smelting reduction process are described by Equations (2)–(15). Gibbs free energy changes of these reactions at different temperatures were calculated using FactSage 6.4, with results shown in Table 2.

Table 2 shows alumina, silicon oxide, and titanium dioxide do not react with carbon between 1300 °C and 1500 °C. In contrast, sodium oxide in high-iron red mud is reduced to metallic sodium by carbon below 1300 °C (reaction (5)). Owing to the low boiling point of metallic sodium (883 °C), the generated sodium rapidly vaporizes into flue gas at elevated temperatures [30]. This volatilized sodium can be recovered from the smoke dust. At 1300–1500 °C, iron oxides (Fe_2_O_3_, Fe_3_O_4_, and FeO) react with carbon to form CO or CO_2_. Meanwhile, the Gibbs free energy change for iron oxides reacting with C to form CO is more negative at these temperatures, indicating iron oxides are more likely to be reduced by C to form CO [31]. Both Fe_2_O_3_ and Fe_3_O_4_ can be reduced with CO. However, the standard Gibbs free energy change for the reaction of FeO with CO is positive at 1300–1500 °C, indicating FeO cannot be reduced to metallic iron by CO under standard conditions. Meanwhile, at 1300–1500 °C, a carburization reaction occurs, forming low-melting Fe_3_C (melting point of 1227 °C).

### 3.2. Calculation of Mass Fraction of Liquid Phase and Liquidus of Al_2_O_3_-SiO_2_-CaO-Na_2_O Slag System

The three most abundant substances in high-iron red mud are iron oxide, alumina, and silicon oxide. Without calcium oxide addition, the reduced slag primarily comprises high-melting-point alumina and silicon oxide, which negatively affects reduction and metal-slag separation efficiency. Therefore, calcium oxide is required to adjust slag viscosity and fluidity. In smelting reduction processes, basicity (*R*), defined as the ratio of total basic oxide mass percentage to total acidic oxide mass percentage, is widely used to describe slag acidity-alkalinity. For high-iron red mud, the acidic oxides involved include alumina and silicon oxide. The ternary basicity formula for high-iron red mud is defined as follows.

(16) $$R=\frac{w_{\mathrm{CaO}}}{w_{{\mathrm{Al}}_{2}O_{3}}+w_{{\mathrm{SiO}}_{2}}}$$

where *w*_CaO_ is the mass percentage of CaO, wt.%; *w*_Al2O3_ is the mass percentage of Al_2_O_3_, wt.%; *w*_SiO2_ is the mass percentage of SiO_2_, wt.%.

The alumina industry commonly uses aluminum-silicon ratio (the mass ratio of alumina to silicon oxide, A/S) and calcium-silicon ratio (the mass ratio of calcium oxide to silicon oxide, C/S) to characterize bauxite and red mud quality. The basicity formula can be expressed using these ratios, yielding the following Equation.

(17) $$R=\frac{C/S}{A/S+1}$$

Equation (17) indicates that the basicity of reduction slag correlates with A/S and C/S, so A/S and C/S should be considered when calculating the slag’s liquid phase mass fraction and liquidus.

#### 3.2.1. Effect of Different Temperatures and Sodium Oxide Contents on Calculation Results

**Figure 4:**
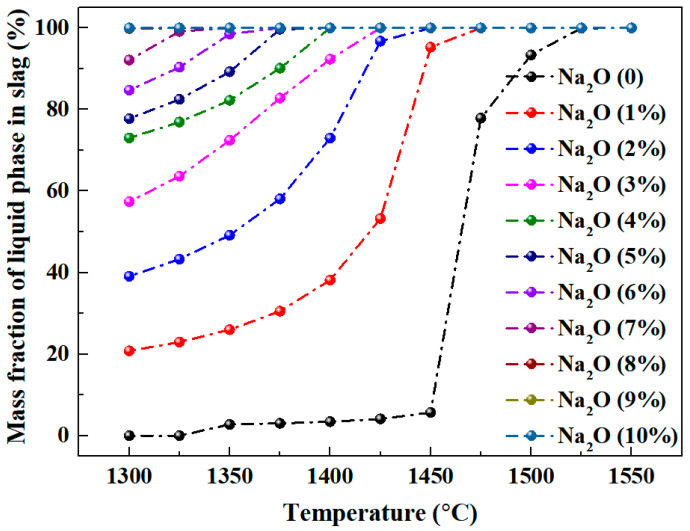
Mass fraction of liquid phase in Al_2_O_3_-SiO_2_-CaO-Na_2_O slag with different temperatures and sodium oxide contents.

Figure 4 shows the calculation results of liquid phase mass fractions in Al_2_O_3_-SiO_2_-CaO-Na_2_O slag under varying temperatures and Na_2_O contents, calculated using the Equilib module of FactSage 6.4 with A/S = 2 and C/S = 3.

In the Al_2_O_3_-SiO_2_-CaO slag without Na_2_O, the liquid phase mass fraction is notably low at 5.69% below 1450 °C. Upon heating to 1475 °C, it increases rapidly to 77.88%, and further increases to 99.92% at 1525 °C. This indicates that the absence of sodium oxide increases the melting point of slag, requiring a reduction temperature exceeding 1500 °C for enhancing fluidity. With increasing sodium oxide content, the liquid phase mass fraction of the slag increases notably. At 1300 °C, it rises sharply from 20.79% (1% sodium oxide) to 99.85% (8% sodium oxide). This suggests that under constant alumina, silicon oxide, and calcium oxide, increasing sodium oxide alone can significantly enhance the slag’s liquid phase mass fraction. This increase in the liquid phase thus enhances slag fluidity. At constant sodium oxide content, the liquid phase mass fraction of the slag increases gradually with temperature. Specifically, in Al_2_O_3_-SiO_2_-CaO-Na_2_O slag with 1% Na_2_O, the liquid phase mass fraction reaches 95.28% at 1450 °C. Further heating to 1475 °C achieves the liquid phase mass fraction of 100%.

**Figure 5:**
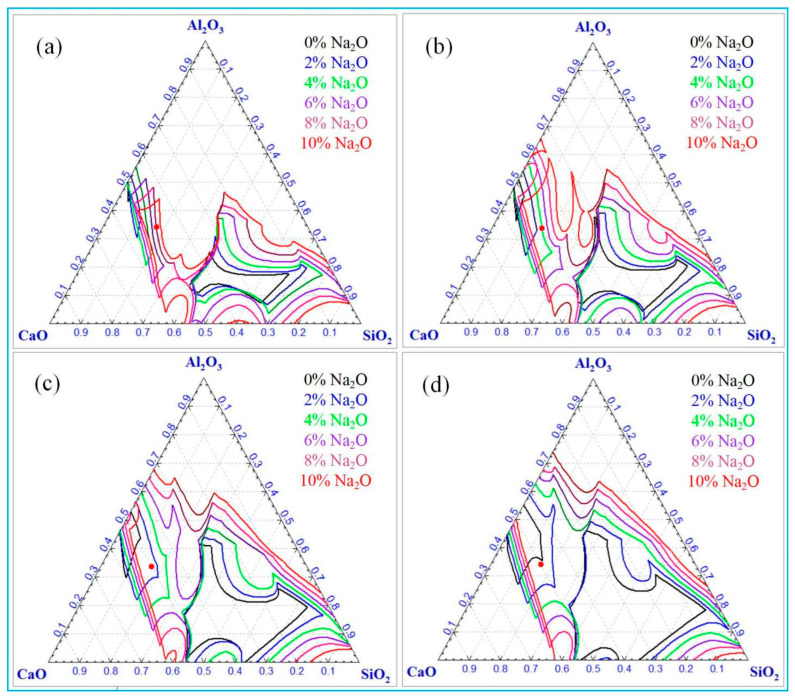
Liquidus of Al_2_O_3_-SiO_2_-CaO-Na_2_O slag with different temperatures and sodium oxide contents. (**a**) 1350 °C, (**b**) 1400 °C, (**c**) 1450 °C, (**d**) 1500 °C.

Figure 5 shows the effects of different temperatures (1350–1500 °C) and sodium oxide concentrations on the liquidus of Al_2_O_3_-SiO_2_-CaO-Na_2_O slag. Computations were performed using the Phase Diagram of FactSage 6.4, with a fixed A/S ratio of 2 and C/S ratio of 3.

Figure 5 shows that at constant sodium oxide content, the slag’s liquidus region expands gradually with increasing temperature, indicating that higher temperatures significantly promote slag liquid phase formation. Without sodium oxide, two distinct liquid phase zones are predicted. Notably, when the A/S ratio is 2 and the C/S ratio is 3, the specific slag composition (marked by the red dot in the figure) remains clearly distant from the liquidus below 1500 °C, resulting in a lower liquid phase mass fraction. This is in consonance with the result in Figure 4. Conversely, at 1500 °C, this point is close to the liquidus, indicating a high liquid phase mass fraction at this temperature. At a constant temperature, the liquidus range of slag progressively expands with increasing sodium oxide content, indicating a gradual rise in the liquid phase mass fraction. Specifically, at 1350 °C, full liquefaction (100% liquid phase) occurs at 7% Na_2_O. This requirement decreases to 4% at 1400 °C, 2% at 1450 °C, and only 1% at 1500 °C. These data reveal a non-linear correlation between temperature and Na_2_O content in regulating the slag’s liquid phase mass fraction. This calculation highlights the significant impact of temperature and the key role of Na_2_O in regulating slag rheological properties. Notably, with constant alumina, silica, and calcium oxide contents, increasing Na_2_O content enhances the liquid phase in the slag. Analysis of Figure 5 data shows Na_2_O content variations significantly affect liquidus boundaries, indicating slag fluidity is strongly correlated with Na_2_O concentration. Thus, adjusting Na_2_O content effectively controls slag fluidity in metallurgical processes. Additionally, Na_2_O is a key regulator of phase equilibrium in this temperature range.

#### 3.2.2. Effect of Different C/S and Temperature on Calculation Results

**Figure 6:**
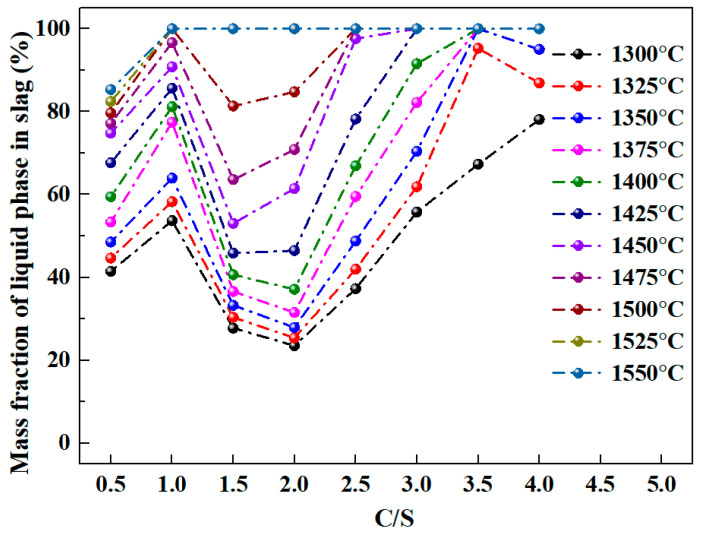
Liquid content in Al_2_O_3_-SiO_2_-CaO-Na_2_O slag with different C/S and temperature.

Figure 6 shows the liquid phase mass fraction in the Al_2_O_3_-SiO_2_-CaO-Na_2_O slag under different C/S ratios and temperatures, with calculations conducted under a fixed 3% Na_2_O content and an A/S ratio of 2.

Figure 6 shows that the liquid phase mass fraction in slag increases gradually with temperature rise. At 1300 °C, the 4 C/S ratio slag peaks at 78.09% liquid phase. At 1350 °C, the 3.5 C/S ratio slag reaches 100% liquid phase, while the 4 C/S ratio slag decreases to 95.01%. Further temperature elevation enables 100% liquid phase even at low C/S ratios, specifically, the 1 C/S ratio slag achieves full liquefaction at 1500 °C. Notably, higher temperatures correspond to increased energy consumption. Under constant temperature, the slag’s liquid phase mass fraction shows a distinct trend with varying C/S ratios: first increasing, then decreasing, and finally increasing again. Within 1300–1400 °C, the liquid phase mass fraction decreases and reaches its minimum at a C/S ratio of 2, while within 1425–1500 °C, the minimum occurs at a C/S ratio of 1.5. At a C/S ratio of 2.5, the slag requires 1475 °C to achieve 100% liquid phase mass fraction. Notably, increasing the C/S ratio to 3 lowers the required temperature to 1425 °C. However, a C/S ratio of 3.5 requires a higher temperature of 1350 °C. Thus, C/S ratio variations significantly affect the temperature dependence of the slag’s liquid phase mass fraction.

**Figure 7:**
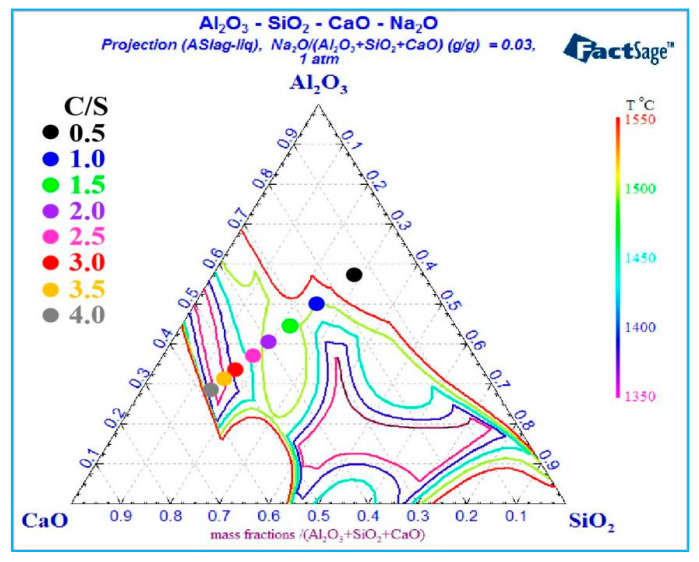
Liquidus of Al_2_O_3_-SiO_2_-CaO-Na_2_O slag with different C/S.

Figure 7 shows the liquidus behavior within the Al_2_O_3_-SiO_2_-CaO-Na_2_O quaternary slag system under varying temperatures and C/S ratios, with a fixed sodium oxide content of 3% and an A/S ratio of 2.

Figure 7 shows that below 1500 °C, the phase diagram shows two distinct liquid phase zones. Above 1500 °C, these zones merge into one, indicating that elevated temperatures promote liquid phase zone convergence. Additionally, the expanding liquidus-enclosed region with rising temperature reflects a gradual increase in the slag’s liquid phase mass fraction. As the C/S ratio increases from 0.5 to 2, the slag composition point first approaches the 1550 °C liquidus (closely aligning with the 1500 °C liquidus) before gradually deviating from the 1500 °C liquidus. This indicates the slag’s liquid phase mass fraction first rises, then declines, and reverses to increase when the C/S ratio exceeds 2, aligning with the trend in Figure 6. As the C/S ratio increases from 2.5 to 3.5, the slag composition point approaches the 1450 °C liquidus, intersecting it at a C/S ratio of 3, then nearing the 1400 °C liquidus. At a C/S ratio of 3.5, the slag composition point coincides exactly with the 1350 °C liquidus, where the liquid phase mass fraction reaches 100%. Further increasing the C/S ratio causes the slag composition point to deviate from the 1350 °C liquidus region, demonstrating that C/S ratio variations significantly affect the slag composition point and its correlation with liquidus temperatures.

## 4. Results and Discussion

### 4.1. Reduction Behavior of High-Iron Red Mud Under Different Experimental Parameters in Smelting Reduction

**Figure 8:**
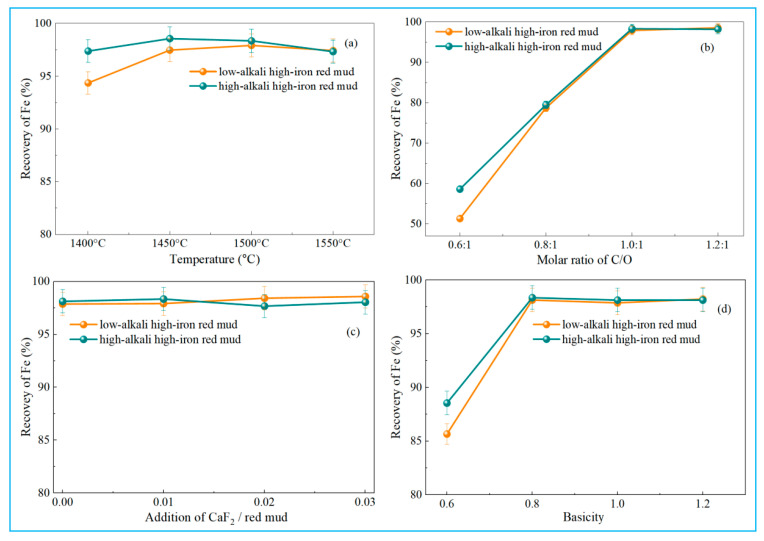
Effect of different experimental parameters on recovery of iron. (**a**) C/O of 1:1, basicity of 1, and 1% CaF_2_, (**b**) 1500 °C, basicity of 1, and 1% CaF_2_, (**c**) 1500 °C, C/O of 1:1, and basicity of 1, (**d**) 1500 °C, C/O of 1:1, and without CaF_2_.

To investigate the effect of temperature on the reduction behavior of high-iron red mud, smelting reduction experiments were conducted under controlled conditions. Reduction temperatures ranged from 1400 °C to 1550 °C (50 °C increments), with a carbon-oxygen molar ratio (defined as carbon in reductant to oxygen in hematite, C/O) of 1:1, basicity of 1, and 1% calcium fluoride (relative to red mud mass) added. Iron recoveries at different temperatures are shown in Figure 8a.

As shown in Figure 8a, iron recovery in high-iron red mud first increased, then slightly decreased with temperature. Iron primarily existed as highly reducible hematite. Specifically, at 1400 °C, low-alkali high-iron red mud achieved 94.36% iron recovery, while high-alkali samples showed higher recovery at 97.38%. Iron recovery of low-alkali high-iron red mud rose to 97.47% at 1450 °C and peaked at 97.92% at 1500 °C, while that of high-alkali samples peaked at 98.56% at 1450 °C. Low-alkali high-iron red mud contains 3.02% Na_2_O, whereas high-alkali samples contain 6.48% Na_2_O. Consequently, at 1400 °C, low-alkali high-iron red mud showed slightly lower iron recovery, demonstrating the role of Na_2_O in enhancing iron recovery. As temperature rises, iron recoveries in both low-alkali and high-alkali high-iron red mud samples decrease slightly. This may be due to slag splashing at high temperatures, which entrains some iron into slag, causing a minor recovery drop. Figure 8a confirms the feasibility of smelting reduction for iron recovery from high-iron red mud; recovery exceeds 97% at 1450 °C, demonstrating process effectiveness. Additionally, higher Na_2_O content in high-iron red mud positively impacts the reduction process.

Figure 8b shows the effect of C/O ratio on iron recovery during smelting reduction with experimental parameters of 1500 °C, basicity 1, and 1% CaF_2_ addition. With increasing C/O ratio, iron recovery gradually rose to a peak at 1:1, then stabilized. Hematite (Fe_2_O_3_) reduction with carbon occurs via two primary mechanisms: CO generation requires a 1:1 C/O ratio, while CO_2_ generation requires 0.5:1. Figure 8b shows that iron recovery peaked at a C/O ratio of 1:1 and remained stable as the ratio continued to increase. At C/O = 1:1, iron recovery reached 97.92% (low-alkali high-iron red mud) and 98.05% (high-alkali high-iron red mud), indicating complete iron reduction and separation. Experimental results align with theoretical predictions, confirming that maintaining a C/O ratio of at least 1:1 is a key parameter for improving iron recovery from high-iron red mud.

Figure 8c shows the effect of calcium fluoride addition on iron recovery during smelting reduction with a temperature of 1500 °C, C/O ratio of 1:1, and a basicity of 1. Iron recovery fluctuated around 98% as calcium fluoride addition increased from 0 to 3%, indicating a minimal influence of the dosage of CaF_2_. Due to the high Na_2_O content in high-iron red mud, the reduction slag maintains good fluidity. Adding CaF_2_ minimally improves slag fluidity, thus having little effect on iron recovery. Consequently, CaF_2_ was omitted in subsequent reductions for economic and environmental reasons.

Figure 8d shows the effect of basicity on iron recovery during smelting reduction with a temperature of 1500 °C, C/O ratio of 1:1, and no calcium fluoride added. For high-iron red mud, increasing the basicity from 0.6 to 0.8 improved iron recovery. The iron recovery of low-alkali high-iron red mud rose from 85.67% to 98.14% and that of high-alkali high-iron red mud increased from 88.55% to 98.36%. Further increasing basicity beyond 0.8 showed no significant recovery improvement. With the basicity increased from 0.6 to 0.8, the C/S ratio of low-alkali high-iron red mud increased from 2.27 to 3.03, while that of high-alkali high-iron red mud increased from 1.65 to 2.2.

Figure 6 shows that at a constant A/S ratio, increasing the C/S ratio enhances the liquid phase mass fraction of slag at 1500 °C and reduces viscosity. Raising basicity from 0.6 to 0.8 improved both high-iron red mud samples by increasing reduced slag liquid phase fraction, lowering viscosity, and thus boosting iron recovery efficiency. At basicity = 1, the C/S ratios of the reduced slag were 3.78 (low-alkali high-iron red mud) and 2.75 (high-alkali high-iron red mud), respectively, with 100% liquid phase mass fraction achieved at 1500 °C. Further basicity increase had minimal effect on iron recovery, thereby identifying a basicity of 0.8 as the optimal condition for achieving maximum iron recovery. Notably, these improvements occurred without calcium fluoride addition, demonstrating that elevated basicity independently enhances iron recovery even in the presence of Na_2_O. Therefore, basicity regulation is critical for optimizing high-iron red mud smelting reduction.

Figure 8 shows the optimal smelting reduction conditions for high-iron red mud: reduction temperature of 1500 °C, C/O ratio of 1:1, basicity of 0.8, and no calcium fluoride addition. Under these conditions, iron recoveries reached 98.14% (low-alkali) and 98.36% (high-alkali) for high-iron red mud samples, which were higher than the 96.63% metallization rate in the investigation work of Feng et al. [1]. The resulting pig iron was polished, and its chemical composition was analyzed by X-ray fluorescence (XRF), with detailed results in Table 3.

Table 3 shows that the carbon content of pig iron ranges from 4.19% to 4.32%. Trace amounts of Al and Si were detected, indicating Al_2_O_3_ and SiO_2_ remained inert during smelting reduction. During smelting reduction, P_2_O_5_ contained in high-iron red mud can be easily reduced to phosphorus by carbon. Due to a low boiling point (280 °C), part of phosphorus evaporates from molten iron, during which phosphorus easily combines with metallic iron to form Fe_3_P and Fe_2_P [28]. Therefore, a small amount of phosphorus being discharged, most of the phosphorus remains in the reduced molten iron. In this study, a certain amount of calcium oxide was added to adjust the alkalinity, thereby playing a role in the dephosphorization of molten iron to a certain extent [33]. With extremely low S and P contents, the pig iron is suitable for direct steel-making use, meeting the L03 industrial standard for steel-making pig iron. The total iron content in pig iron obtained in this work is lower than Guo’s research results [28], while the sulfur content (<0.025%) is also lower than what can be used for steelmaking.

**Figure 9:**
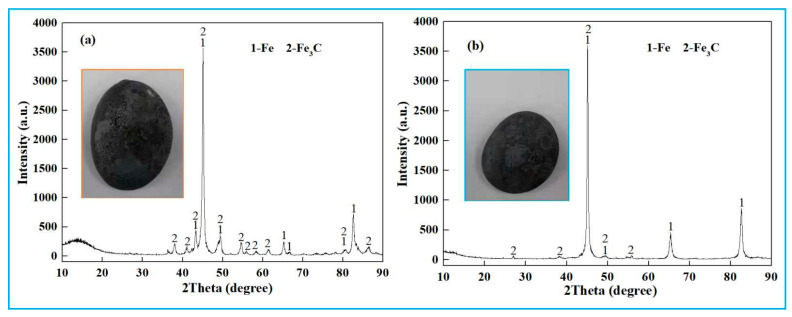
XRD of the reduced iron. (**a**) low-alkali high-iron red mud, (**b**) high-alkali high-iron red mud.

**Figure 10:**
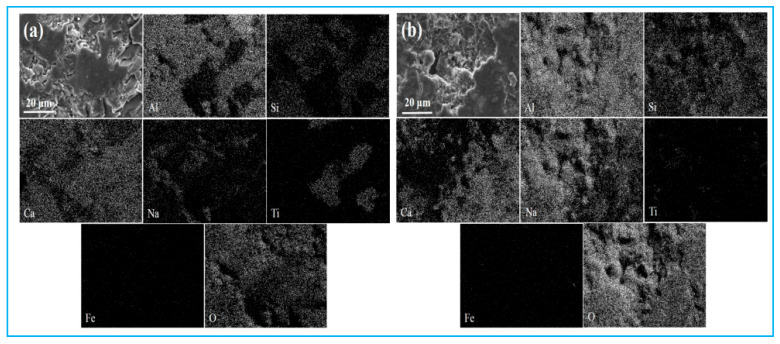
SEM-EDS results of the reduced slag. (**a**) low-alkali high-iron red mud, (**b**) high-alkali high-iron red mud.

Reduced pig iron samples from low-alkali and high-alkali high-iron red mud were sanded and polished for phase analysis. The XRD results (Figure 9) show that the main phases are Fe and Fe_3_C. Fe_3_C lowers the melting point of the molten iron, facilitating its separation from slag during metallurgical processes. The main components and SEM-EDS results of the reduction slag are shown in Table 4 and Figure 10. The main components of reduction slag are alumina, silicon oxide, calcium oxide, titanium oxide, etc., which are the main components of building materials.

### 4.2. Kinetic Analysis of Recovery of Iron from High-Iron Red Mud During Smelting Reduction

**Figure 11:**
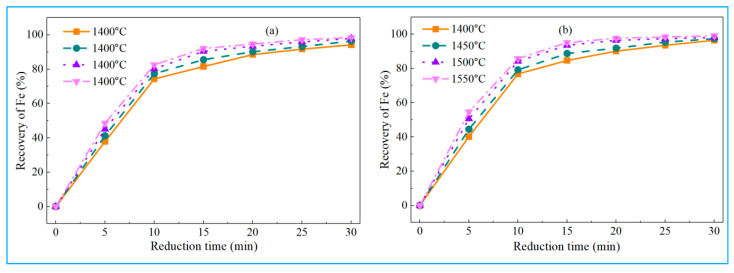
Effect of reduction time on iron recovery rate. (**a**) low-alkali high-iron red mud, (**b**) high-alkali high-iron red mud.

Figure 11 shows iron recovery results for low-alkali and high-alkali high-iron red mud under different reduction temperatures (1400–1550 °C at 50 °C intervals) and reduction times.

Figure 11a shows the iron recovery trend of low-alkali high-iron red mud with varying reduction time and temperature. Initially, iron recovery rose rapidly with time, then increased gradually, while temperature elevation also promoted recovery. At 1400 °C, iron recovery rose from 37.89% to 94.23% as the reduction time increased from 5 min to 30 min. At 1550 °C, iron recovery was 98.43% with a reduction time of 30 min. Notably, iron recovery slowed with further time extension. Figure 11b shows that for high-alkali high-iron red mud, iron recovery increased rapidly, then slowly with increasing reduction time, and also increased gradually with temperature. At 1400 °C, iron recovery rose from 40.26% to 96.47% as the reduction time increased from 5 min to 30 min. At 1550 °C, iron recovery was 99.15% with a reduction time of 30 min. Notably, under identical conditions, high-alkali high-iron red mud showed higher recovery than low-alkali high-iron red mud due to sodium oxide reducing slag viscosity (Na_2_O provides O^2−^ ions to depolymerize the polymerized silicate tetrahedra [34]), improving fluidity, and facilitating reduction.

In addition, CO by-products stirred the molten pool, enhancing the mixing of reactants. The reduction process of both high-iron red mud samples can be divided into two stages: 0–10 min (initial stage) and 15–30 min (subsequent stage).

**Figure 12:**
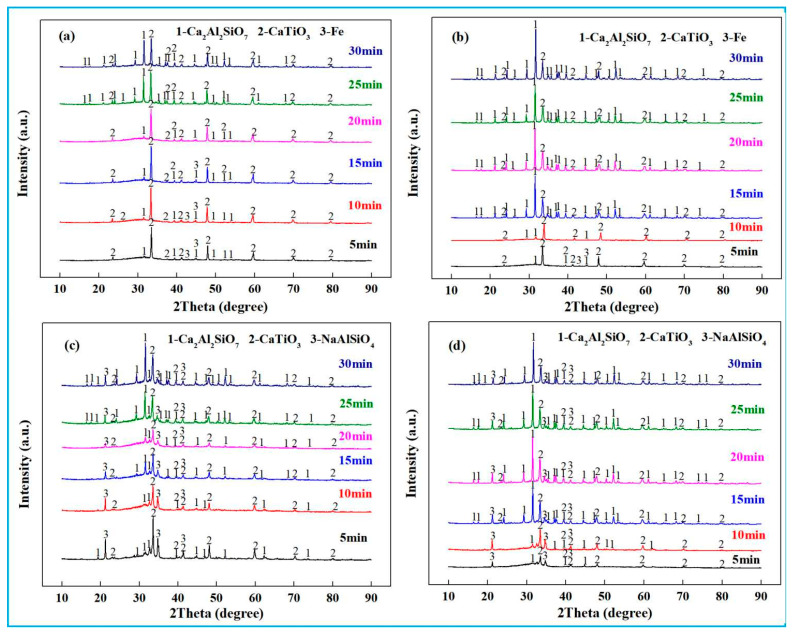
XRD of the reduced slag. (**a**) reduced slag of low-alkali high-iron red mud at 1400 °C, (**b**) reduced slag of low-alkali high-iron red mud at 1500 °C, (**c**) reduced slag of high-alkali high-iron red mud at 1400 °C, (**d**) reduced slag of high-alkali high-iron red mud at 1500 °C.

Figure 12a,b shows XRD results of low-alkali high-iron red mud reduction slag at 1400 °C and 1500 °C, respectively. At 1400 °C (Figure 12a), gehlenite and calcium titanate were the dominant phases after reduction [1]. Shorter reduction time led to slightly higher slag viscosity, limiting iron melt settling and leaving unseparated metallic iron in slag. This decreased with prolonged reduction, with metallic iron disappearing at 20 min as gehlenite diffraction peaks strengthened. At 1500 °C (Figure 12b), low slag viscosity and good fluidity resulted in unseparated metallic iron only in the initial 5 min reduction, and this vanished by 10 min. The disappearance of metallic iron reflects the composition and structure changes in slag, highlighting the critical role of reduction time in metallurgical processes.

Figure 12c,d show XRD results of high-alkali high-iron red mud reduction slag at 1400 °C and 1500 °C. After smelting reduction, the slag primarily contained gehlenite and calcium titanate, with >6% Na_2_O (existing as nepheline upon cooling [35]). High Na_2_O content is beneficial to the fluidity of the slag, conducive to the efficient separation of molten iron and slag. No diffraction peak of metallic iron was detected. With prolonged reduction time, Al_2_O_3_, SiO_2_, and CaO reacted to form gehlenite. TiO_2_ from high-iron red mud remained in slag, reacting with CaO to form CaTiO_3_. At 1500 °C, the gehlenite peak intensity increased with time, while the diffraction peaks of nepheline decreased. Under high temperature, a portion of Na_2_O was reduced to metallic sodium (boiling point of 883 °C), which volatilized into flue gas.

During the smelting reduction of high-iron red mud, the primary reduction reaction is the carbothermal reduction of hematite, generating CO. In these kinetic experiments, the reductant carbon drives the dominant solid (carbon)-liquid (melt of high-iron red mud and additives) reaction. Based on the solid-liquid reaction model (Equation (18)) [36], the overall reaction rate is governed by both the chemical reaction rate between iron ions and carbon and the metallic iron diffusion rate in the liquid phase. Thus, reaction kinetics complexity depends on interfacial chemical reactions and the diffusion rate of metallic iron.

(18) $$\frac{1}{k}=\frac{1}{k_{d}}+\frac{1}{k_{r}}$$

where *k* is the total reaction rate constant; *k*_d_ is the mass transfer coefficient; *k*_r_ is the rate constant of chemical reaction.

The smelting reduction of high-iron red mud can be simplified into three steps.

(1) Fe^3+^ and O^2−^ ions diffuse to the molten-carbon interface.
(2) At the molten-carbon interface, Fe^3+^ adsorbs on the carbon surface and reacts with carbon to form metallic iron. The lattice oxygen of iron oxides combines with carbon to form CO gas. Iron then carburizes to form Fe_3_C, followed by product desorption.
(3) Fe_3_C(l) and CO(g) overcome interfacial adsorption and leave the reaction interface.

The entire process is described by Equations (19)–(22).

3(Fe_2_O_3_) + C(s) = 2(Fe_3_O_4_) + CO(g) (19)

(Fe_3_O_4_) + C(s) = 3(FeO) + CO(g) (20)

(FeO) + C(s) = Fe(s) + CO(g)(21)

3Fe(s) + C(s) = Fe_3_C(l) (22)

From Figure 11, during 0–10 min, iron recovery from high-iron red mud increases significantly with a nearly linear trend, indicating the reduction is governed by interfacial chemical reactions rather than oxide concentration. In the later stage (15~30 min), the growth of iron recovery slows down, suggesting diffusion control dominates. Thus, the smelting reduction process of high-iron red mud can be divided into two stages: interfacial reaction control (0~10 min) and diffusion control (15~30 min), and is effectively described by a shrinkage core model.

In the initial reaction stage (0~10 min), reaction kinetics is primarily governed by interfacial chemical processes, with the kinetics equation described by Equation (23) [24].

(23) $$1-{\left(1-x\right)}^{1/3}=k_{r}t$$

where *k*_r_ is the rate constant of chemical reaction, s^−1^; *t* is the reduction time, s; *x* is the recovery of iron, %.

In the second reaction stage (15~30 min), the kinetic equation under diffusion-controlled restrictive conditions is given by Equation (24) [37].

(24) $$1-\frac{2}{3}x-{\left(1-x\right)}^{2/3}=k_{d}t$$

where *k*_d_ is the mass transfer coefficient, s^−1^; *t* is reduction time, s; *x* is recovery of iron, %.

The apparent activation energy of chemical reactions during the smelting reduction of high-iron red mud can be calculated using the Arrhenius equation.

(25) lnk=−E/RT+lnA

where *k* is the reaction rate constant, s^−1^; *E* is the apparent activation energy of the chemical reaction, kJ·mol^−1^; *R* is the ideal gas constant, J·mol^−1^·K^−1^; *T* is the temperature, K; *A* is the chemical reaction frequency factor, s^−1^.

**Figure 13:**
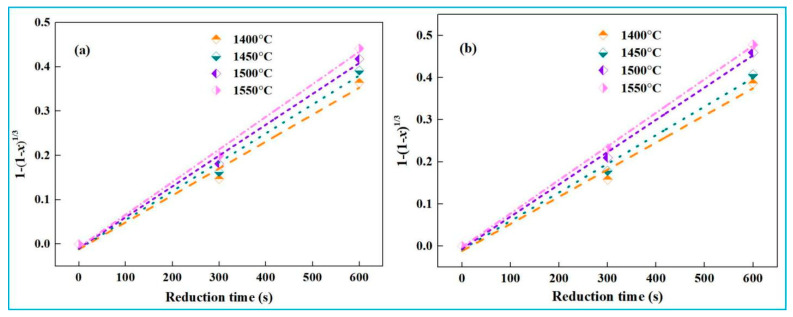
Numerical fitting of iron recovery rate and time (0~10 min). (**a**) low-alkali high-iron red mud, (**b**) high-alkali high-iron red mud.

Based on Figure 11, the numerical fitting relationships between iron recovery and reduction time during the initial reaction stage (0~10 min) for both high-iron red mud samples were calculated using Equation (23), with corresponding plots shown in Figure 13.

**Figure 14:**
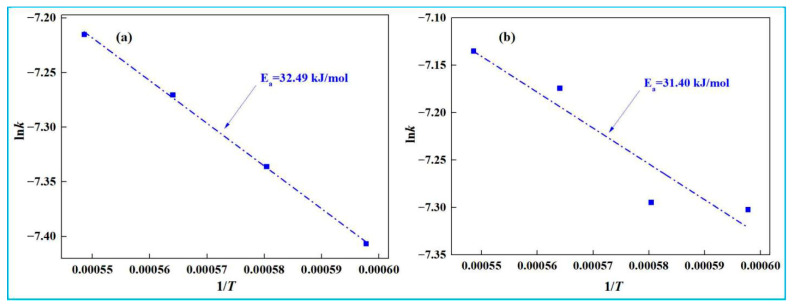
Relationship between ln*k* and 1/*T* (0~10 min). (**a**) low-alkali high-iron red mud, (**b**) high-alkali high-iron red mud.

Based on the fitting curve of 1 − (1 − *x*)^1/3^ and time *t* in Figure 13, the reaction rate constants (*k*) at different temperatures were determined. A subsequent ln*k* and 1/*T* plot was constructed (Figure 14), enabling the analysis of the temperature dependence of reaction rate constants.

Figure 14 shows that the slope of the ln*k* and 1/*T* fitting line is steeper for low-alkali high-iron red mud than for high-alkali high-iron red mud. According to Equation (25), the slope equals −*E*/R, enabling the calculation of apparent activation energy (*E*). For the initial stage (0~10 min), the *E* values of low-alkali high-iron red mud and high-alkali high-iron red mud during smelting reduction are 32.49 kJ·mol^−1^ and 31.40 kJ·mol^−1^, respectively. Lower *E* indicates a faster reaction rate. Thus, high-alkali high-iron red mud presents quicker reaction due to its higher Na_2_O content, which reduces melt viscosity, improves fluidity, and enhances mass/heat transfer during reaction.

**Figure 15:**
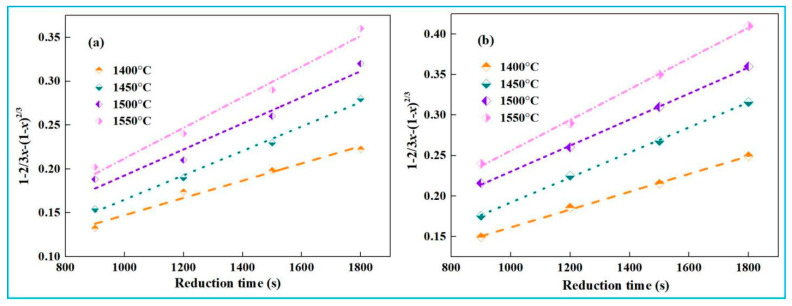
Numerical fitting of iron recovery rate and time (15~30 min). (**a**) low-alkali high-iron red mud, (**b**) high-alkali high-iron red mud.

During the second stage (15~30 min) of smelting reduction, the reaction is diffusion-controlled. Based on the results of Figure 11, numerical fitting relationships between iron recovery and reduction time in this stage were calculated using Equation (24) for high-iron red mud, as shown in Figure 15.

**Figure 16:**
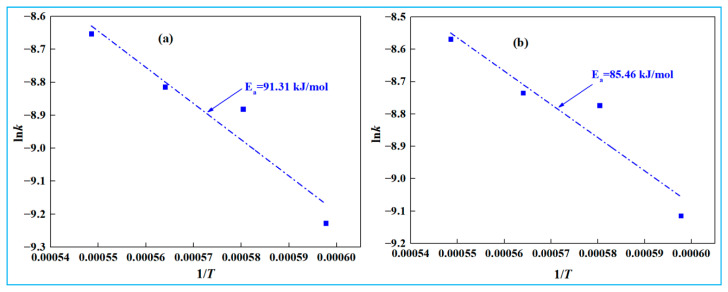
Relationship between ln*k* and 1/*T* (15~30min). (**a**) low-alkali high-iron red mud, (**b**) high-alkali high-iron red mud.

Based on the fitting curve of 1−2/3*x*−(1−*x*)^2/3^ and time *t* shown in Figure 15, reaction rate constants (*k*) at different temperatures were determined. A plot of ln*k* and 1/*T* was then plotted in Figure 16.

Based on Figure 15, during the second stage (15~30 min), the apparent activation energies of low-alkali high-iron red mud and high-alkali high-iron red mud reduction reactions in the smelting reduction process are 91.31 kJ·mol^−1^ and 85.46 kJ·mol^−1^, respectively.

The solid-phase direct reduction of carbon-containing pellets from high-iron red mud is controlled by the mixing of carbon gasification and gas diffusion. The apparent activation energies are 126.48 kJ·mol^−1^ and 119.77 kJ·mol^−1^, respectively [38], which are significantly higher than the apparent activation energy of the smelting reduction process, indicating that the reaction rate of the smelting reduction process of high-iron red mud is relatively quick.

### 4.3. Pilot Scale Experiment of Smelting Reduction of High-Iron Red Mud

#### 4.3.1. Smelting Reduction of High-Iron Red Mud

In this section, a pilot-scale smelting reduction experiment on high-iron red mud was conducted, providing relevant data for the industrial large-scale application of the process.

**Figure 17:**
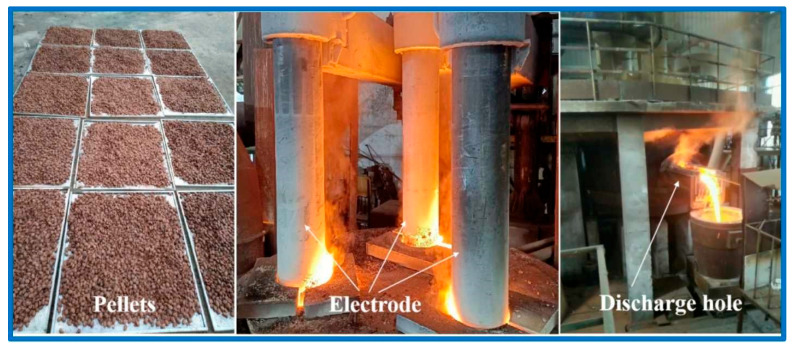
Pilot-scale experiment of smelting reduction of high-alkali high-iron red mud.

First, high-alkali high-iron red mud, reducing agent, and lime were thoroughly mixed in preset proportions. Then, the mixed powder was compacted with a double-roll press into 25 mm × 30 mm pellets. Next, the pressed pellets were dried. A pilot-scale smelting reduction experiment on the pellets was conducted in a 10-ton electric arc furnace, with the process and observed phenomena shown in Figure 17.

**Figure 18:**
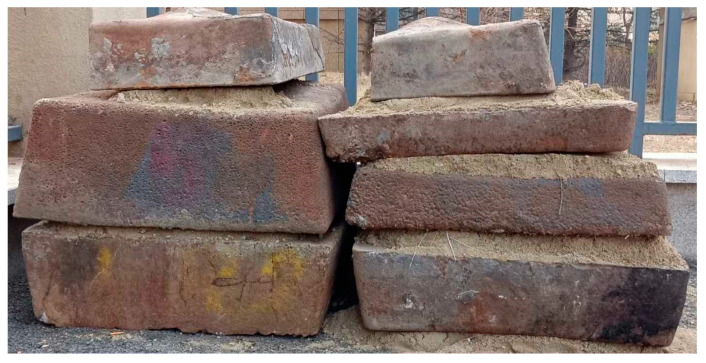
Photograph of the reduced metal from high-alkali high-iron red mud.

After the smelting reduction experiment, the power was turned off. Molten iron and reduction slag were discharged into a slag ladle via the arc furnace’s bottom outlet, then cast into a mold. During casting, the reduction slag showed excellent flowability with extremely low viscosity. Molten iron and slag stratified clearly due to density differences, with iron settling naturally. After complete cooling, the mold was inverted to separate the metal and slag. The metal block (Figure 18) has a smooth surface, visually confirming the successful reduction.

The chemical composition analysis of the reduced metal from high-alkali high-iron red mud (results shown in Table 5) reveals significantly low phosphorus and sulfur contents, meeting the L03 industrial standard for steel-making pig iron. Thus, the reduced pig iron can be directly used as a steel-making material.

#### 4.3.2. Utilization Strategy of Reduction Slag

During the smelting reduction of high-iron red mud, besides yielding raw pig iron suitable for direct steel-making, molten reduction slag (mainly composed of Al_2_O_3_, SiO_2_, and CaO) is produced. This slag can be utilized via two distinct pathways.

(1) Casting and Controlled Cooling: Slag is cast and slow-cooled under controlled conditions, facilitating cutting into standardized roadside stones with improved mechanical durability.

**Figure 19:**
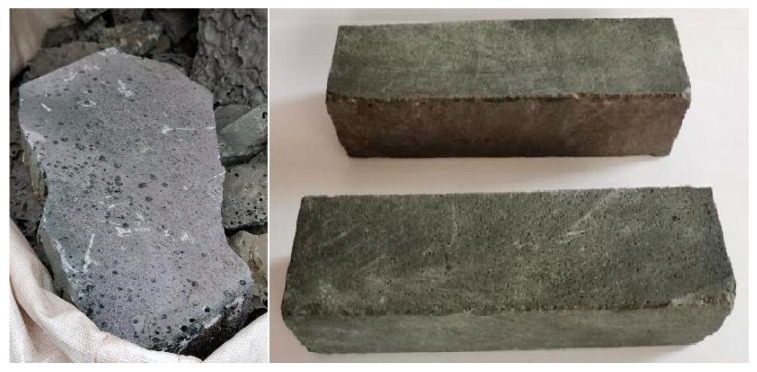
Photograph of the reduced slag block and samples cut from reduced slag.

Representative samples of as-cast slag blocks and sectioned specimens are shown in Figure 19. Post-treatment, the reduced slag showed a homogeneous, compact morphology with minimal porosity. Mechanical performance was quantified via uniaxial compression and three-point bending tests on standardized sectioned specimens (detailed in Table 6). The reduced slag blocks show a compressive strength of 102 MPa and flexural strength of 12.5 MPa, confirming their suitability for roadside stone applications.

(2) Water Quenching: Direct water quenching of molten slag produces vitrified granulated water-quenched slag, which, after drying and pulverization, acts as a precursor for low-carbon cement clinker synthesis without extra calcination.

**Figure 20:**
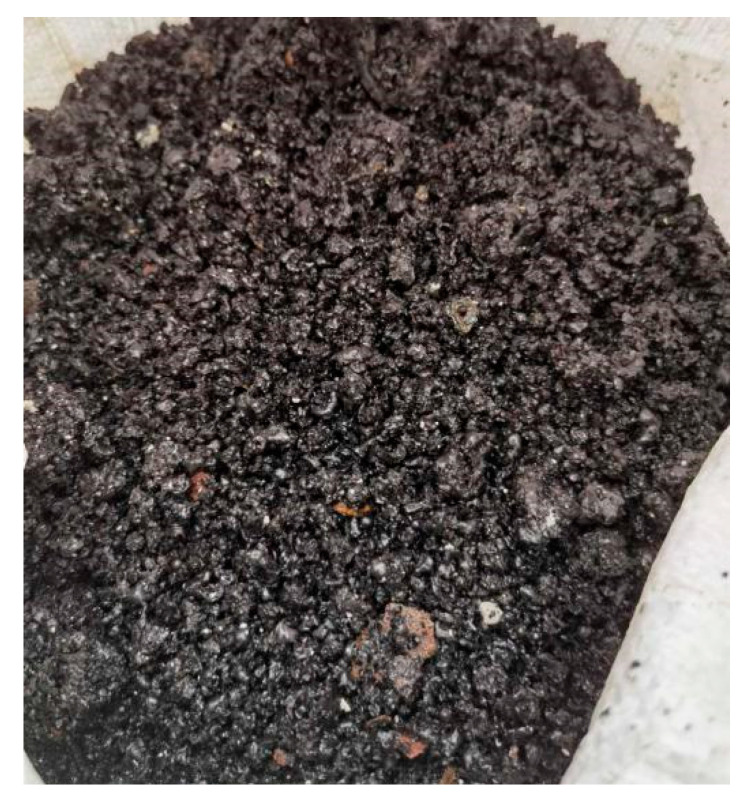
Photograph of water-quenched slag.

Direct water quenching of molten reduction slag effectively retains high vitreous phase content (>95%) in the water-quenched slag (Figure 20). The water-quenched slag shows excellent reactivity, with a 28-day activity index >95% shown in Table 7, comparable to commercial S95-grade ground granulated blast furnace slag (GGBS). After drying and pulverization, it can substitute 30–50% of cement formulations, facilitating low-carbon clinker production with ~500 kg CO_2_ reduction per ton of clinker.

Although sodium oxide can significantly reduce the melting point of the reduction slag and promote its fluidity during the smelting reduction of high-iron red mud, it will be reduced to metallic sodium and volatilize into the flue gas, which causes erosion of the furnace lining. Therefore, in order to reduce the erosion of the furnace lining and extend its service life, in the future industrial practical application process, the calcification de-alkalization method can be adopted for the pretreatment of high-iron red mud, reducing the sodium oxide in high-iron red mud to less than 1% [39]. This not only slows down the erosion of the furnace lining but also reduces the impact of sodium oxide on the use of reduction slag in the building materials field.

## 5. Conclusions

This study focuses on the reduction behavior and kinetic analysis of iron recovery from high-iron red mud during smelting reduction. Smelting reduction of high-iron red mud was used to prepare steelmaking pig iron, achieving high-value utilization of high-iron red mud. The main innovative conclusions are as follows:

(1) Thermodynamic analysis indicates that during smelting reduction, alumina, silicon oxide, and titanium dioxide in high-iron red mud do not undergo reduction at 1300–1500 °C. Both hematite and sodium oxide are reducible, with hematite’s carbothermal reduction following the reaction Fe_2_O_3_ + 3C = 2Fe + 3CO.
(2) For Al_2_O_3_-SiO_2_-CaO-Na_2_O slag, increasing temperature and sodium oxide content both enhance the liquid phase mass fraction. Higher sodium oxide content reduces slag viscosity. At constant temperature and A/S, the liquid phase mass fraction first increases, then decreases, and rises again with increasing C/S.
(3) Optimizing reduction temperature, C/O ratio, and basicity significantly improves iron recovery from high-iron red mud, whereas CaF_2_ has minimal impact. Under optimal conditions, iron recoveries of low-alkali high-iron red mud and high-alkali high-iron red mud reached 98.14% and 98.36%, respectively. The reduced pig iron meets the L03 industrial standard for steel-making pig iron.
(4) Kinetic experiments show that the smelting reduction process of high-iron red mud is divided into two stages. In the initial stage (0–10 min), reaction kinetics is governed by interfacial chemical reaction, with apparent activation energies of 32.49 kJ·mol^−1^ (low-alkali) and 31.40 kJ·mol^−1^ (high-alkali), high-iron red mud. In the second stage (15–30 min), diffusion controls the process, with corresponding apparent activation energies of 91.31 kJ·mol^−1^ and 85.46 kJ·mol^−1^, respectively.
(5) Pilot-scale reduction experiments confirm the high-value utilization potential of high-iron red mud for iron recovery. The reduced slag can be used as the roadside stone or cement clinker, with results providing theoretical guidance and technical support for its large-scale and high-value comprehensive utilization.

## Figures and Tables

**Table 1:** Main chemical compositions of high-iron red mud (wt.%).

Compositions	Al_2_O_3_	SiO_2_	Fe_2_O_3_	TiO_2_	Na_2_O	CaO	S	P	LOI	A/S
low-alkali high-iron red mud	17.01	6.11	53.71	6.49	3.02	0.67	0.11	0.13	11.92	2.78
high-alkali high-iron red mud	17.74	10.13	41.63	8.45	6.48	1.60	0.16	0.12	12.56	1.75

**Table 2:** Possible carbothermal reduction reactions of high-iron red mud.

Reaction	ΔG^θ^ (kJ/mol)			Number
	T = 1300 °C	T = 1400 °C	T = 1500 °C	
Al_2_O_3_ + 3C = 2Al + 3CO	421.89	363.67	305.67	(2)
SiO_2_ + 2C = Si + 2CO	132.17	98.24	61.83	(3)
TiO_2_ + 2C = Ti + 2CO	160.97	126.30	91.75	(4)
Na_2_O + C = 2Na + CO	−37.32	−53.86	−70.24	(5)
3Fe_2_O_3_ + C = 2Fe_3_O_4_ + CO	−235.48	−258.24	−280.87	(6)
3Fe_2_O_3_ + 0.5C = 2Fe_3_O_4_ + 0.5CO_2_	−183.62	−197.87	−212.04	(7)
3Fe_2_O_3_ + CO = 2Fe_3_O_4_ + CO_2_	−131.75	−137.51	−143.21	(8)
Fe_3_O_4_ + C = 3FeO + CO	−115.13	−134.93	−158.15	(9)
Fe_3_O_4_ + 0.5C = 3FeO + 0.5CO_2_	−63.26	−74.56	−89.32	(10)
Fe_3_O_4_ + CO = 3FeO + CO_2_	−11.40	−14.20	−20.49	(11)
FeO + C = Fe + CO	−88.80	−103.40	−116.86	(12)
FeO + 0.5C = Fe + 0.5CO_2_	−36.93	−43.04	−48.04	(13)
FeO + CO = Fe + CO_2_	14.94	17.33	20.79	(14)
3Fe + C = Fe_3_C	−5.16	−6.01	−6.55	(15)

**Table 3:** Chemical compositions of reduced metal (wt.%).

Component	Fe	C	Al	Si	Mn	P	S
reduced metal from low-alkali high-iron red mud	94.33	4.32	<0.01	0.32	0.022	0.18	0.025
reduced metal from high-alkali high-iron red mud	94.68	4.19	<0.01	0.16	0.038	0.12	0.024
iron nugget reduced from high-iron red mud in reference [28]	96.52	3.09		0.051	0.013	0.076	0.091
L03 industrial standard for steel-making pig iron (YB/T 5296-2011) [32]		≥3.5	-	≤0.35	≤0.40	>0.10~0.15	≤0.030

**Table 4:** Chemical composition of reduction slag (wt.%).

Component	Al_2_O_3_	SiO_2_	CaO	Na_2_O	TiO_2_	TFe
Reduction slag of low-alkali high-iron red mud	33.92	11.71	34.11	3.06	6.74	0.95
Reduction slag of high-alkali high-iron red mud	36.04	15.81	33.20	6.72	8.19	0.74

**Table 5:** Chemical composition of reduced metal (wt.%).

Component	Fe	C	Al	Si	Mn	P	S
reduced metal from high-alkali high-iron red mud	94.27	4.21	<0.01	0.08	0.025	0.13	0.02
L03 industrial standard for steel-making pig iron (YB/T 5296-2011) [32]		≥3.5	-	≤0.35	≤0.40	>0.10~0.15	≤0.03

**Table 6:** Mechanical properties of the samples cut from reduced slag.

Test Item	Compressive Strength	Flexural Strength
Result	102 MPa	12.5 MPa

**Table 7:** Activity index and compressive strength of the samples prepared from water-quenched slag.

Doping Amount of Water Quenched Slag in Cement Samples	SSA	*R*_7_	*H*_7_	*R*_28_	*H*_28_
30%	418	35.8	89	48.4	93
	492	39.9	98	50.3	96
	538	42.7	106	57.7	112
50%	418	29.3	74	47.8	93
	492	33.8	84	49.7	97
	538	39.9	99	50.6	98

where SSA is the specific surface area of the water-quenched slag, m^2^·kg^−1^; *R*_7_ and *R*_28_ are the compressive strength of test samples for 7 days and 28 days, MPa; *H*_7_ and *H*_28_ are the activity index of test samples for 7 days and 28 days, %.

## Data Availability

The raw and processed data required to reproduce these results are available upon reasonable request.
